# A protein domain-centric approach for the comparative analysis of human and yeast phenotypically relevant mutations

**DOI:** 10.1186/1471-2164-14-S3-S5

**Published:** 2013-05-28

**Authors:** Thomas A Peterson, DoHwan Park, Maricel G Kann

**Affiliations:** 1 Department of Biological Sciences, University of Maryland, Baltimore County, Baltimore, MD, USA; 2 Department of Mathematics and Statistics, University of Maryland, Baltimore County, Baltimore, MD, USA

## Abstract

**Background:**

The body of disease mutations with known phenotypic relevance continues to increase and is expected to do so even faster with the advent of new experimental techniques such as whole-genome sequencing coupled with disease association studies. However, genomic association studies are limited by the molecular complexity of the phenotype being studied and the population size needed to have adequate statistical power. One way to circumvent this problem, which is critical for the study of rare diseases, is to study the molecular patterns emerging from functional studies of existing disease mutations. Current gene-centric analyses to study mutations in coding regions are limited by their inability to account for the functional modularity of the protein. Previous studies of the functional patterns of known human disease mutations have shown a significant tendency to cluster at protein domain positions, namely position-based domain hotspots of disease mutations. However, the limited number of known disease mutations remains the main factor hindering the advancement of mutation studies at a functional level. In this paper, we address this problem by incorporating mutations known to be disruptive of phenotypes in other species. Focusing on two evolutionarily distant organisms, human and yeast, we describe the first inter-species analysis of mutations of phenotypic relevance at the protein domain level.

**Results:**

The results of this analysis reveal that phenotypic mutations from yeast cluster at specific positions on protein domains, a characteristic previously revealed to be displayed by human disease mutations. We found over one hundred domain hotspots in yeast with approximately 50% in the exact same domain position as known human disease mutations.

**Conclusions:**

We describe an analysis using protein domains as a framework for transferring functional information by studying domain hotspots in human and yeast and relating phenotypic changes in yeast to diseases in human. This first-of-a-kind study of phenotypically relevant yeast mutations in relation to human disease mutations demonstrates the utility of a multi-species analysis for advancing the understanding of the relationship between genetic mutations and phenotypic changes at the organismal level.

## Background

The study of human genomic variations, in particular those in protein coding regions, can lead to new hypotheses about the molecular mechanisms of human diseases and might provide critical knowledge about individual response to therapy [1,2]. The advent of large-scale experimental techniques is providing new phenotypic associations for genomic variations [3-5]. However, genomic association studies are limited by the molecular complexity of the phenotype being studied and the cohort size needed to have adequate statistical power. One way to circumvent this problem, which is critical in the study of rare diseases, is to investigate the molecular patterns emerging from functional studies of existing disease mutations. In current large association studies, such as GWAS or upcoming whole-exome and whole-genome sequencing, this is accomplished by aggregating mutations that disrupt the same gene [6,7], pathway [8], or network [9]. In many cases, these molecular variations associated with human diseases have patterns that are similar to those producing a phenotypic change in other species. For example, the comparison between close species has made significant contributions to the biomedical field, such as the use of mice [10] and rats [11] for genetics and drug discovery. In addition, studies across species with longer evolutionary distances to human have many advantages and could bring new perspectives into the study of molecular mechanisms of human phenotypic variations. For instance, the functional analysis of variations in yeast, an organism that can be easily genetically manipulated, has shed light on variations in their human gene orthologs, as shown in McGary *et al. *[12]. The authors demonstrated the potential of a systematic study of phenotypes produced by variations in human and their orthologs in yeast or other distantly related species, providing novel hypotheses about human diseases, which have already resulted in valuable leads for drug discovery.

The vast majority of studies related to human disease mutations are performed by comparison of whole proteins, which here will be denoted by the genes that encode them. However, these whole-protein approaches are of limited applicability to the study of disease mutations due to the fact that they mostly fail to account for protein modularity. Most proteins contain multiple domains that can be recombined in different arrangements to create proteins with different functions [13-15]. As a consequence, not all protein regions have the same function or produce similar phenotypic changes if disrupted. Thus, the specific location of a particular mutation within the protein could be crucial to understanding the mutation's functional effect. The relevance of studying protein domains in the context of disease was also discussed by Zhong *et al. *[16] in their study of protein interactions and their relation to diseases. The authors showed that mutations resulting in complete loss of the protein product (removal of a node in the network) could be different from those disrupting only a protein region or domain (edgetic perturbations). Furthermore, Zhong *et al*. conclude that these edgetic perturbations can cause clinically distinct phenotypes when disrupting different protein domain regions of the same protein. Thus, a domain-centric study of disease mutations has the potential to differentiate among genomic variations by accounting for protein modularity that would have otherwise been grouped together by whole-protein studies.

To capture the disruption of domains by genetic mutations, we have previously created a database to visualize the aggregation patterns of disease mutations at the protein and domain levels for human genomics data (Domain Mapping of Disease Mutations database (DMDM), freely available at http://bioinf.umbc.edu/dmdm/) [17]. More recently, we have developed a statistical approach, the domain significance score (or DS-Score), for finding significantly mutated positions for individual protein domains [18]. We demonstrated that significant DS-Scores indicate that a mutation at a specific position is highly likely to be a contributor to disease in any protein containing the domain in which the mutations are located. In particular, we have shown that Mendelian disease mutations form clusters at protein domain sites [18]. In addition, results from Yue *et al. *[19], Nehrt *et al. *[20], and Peterson *et al. *[18] have further shown that inherited and somatic cancer mutations cluster at specific sites at the protein domain level. Thus, these studies show how the domain analysis enables the discovery of domain hotspots of mutations with phenotypic relevance by aggregating mutations that share the same domain location but are localized in different genes. However, the discovery of these highly deleterious domain sites by aggregation of mutational data with known phenotypic effect is limited by the availability of such mutational data. As a result, the DS-Score method based on human data has low coverage when analyzing mutations from large-scale sequencing studies. To address this issue, more annotated disease mutation data will need to be incorporated into the analysis, preferably from other species in which the phenotypic effect of putative deleterious mutations could be experimentally tested.

In this paper, we describe the first inter-species analysis of mutations of phenotypic relevance at the protein domain level for human and yeast genomes. We perform the comparison between these species by mapping human and yeast mutations into the corresponding domain sites. Protein domains, such as those defined by CDD [21] and Pfam [22], are protein sequence regions that are highly conserved across distantly related species. For instance, when comparing yeast and human domains, we estimate that 87% of all the protein domains found in yeast are also found in human while, using the Homologene database to compare genes, only 20% of the yeast genes have a human ortholog [23]. Similarly, 58% of the human domains are shared with yeast while only 5% of the human genes have yeast orthologs. Since yeast and human analyses show a significant number of common domains, the protein domain framework facilitates the comparison of a significant number of mutations producing phenotypic changes in both species. Using a domain-centric approach, we show that phenotypically relevant mutations in yeast form hotspots at the protein domain level, and that a significant number of these hotspots map to known human disease mutations. Furthermore, our results show that the feature-based DS-Score, a modification of our statistical method that explicitly incorporates annotation from the protein domain models, was most successful at capturing functional commonalities between human and yeast mutations affecting these organisms' phenotypes.

In summary, the work described in this paper demonstrates that domain-centric, inter-species mutation analyses lead to the identification of new domain sites of relevance to human diseases even when performed among species separated by long evolutionary distances. The patterns of evolutionarily conserved and functional mutations associated with phenotypic changes emerging from this study represent a step towards a new paradigm for the analysis of large-scale genomic studies of human diseases.

## Materials and methods

### Databases

A human protein database containing 54,372 proteins was created with 33,963 proteins from RefSeq [24] and 20,409 proteins from Swiss-Prot [25] downloaded via NCBI's E-utilities [23]. Since the RefSeq and Swiss-Prot databases contain many redundant protein entries, we selected only one representative protein for each unique Entrez gene ID, either the longest Swiss-Prot protein, or the longest RefSeq protein if no Swiss-Prot protein was listed for the gene ID. A database of 6,717 verified and hypothetical open reading frame yeast reference proteins was downloaded from the Saccharomyces Genome Database (SGD) [26] on September 28^th^, 2012 (http://downloads.yeastgenome.org/sequence/S288C_reference/orf_protein/). The Homologene database [23] was downloaded from NCBI's FTP site on September 12^th^, 2011. A protein domain set was obtained from the Conserved Domain Database (CDD version 2.25) [21], which includes domains from CDD and the SMART [27], COG [28], and Pfam [22] databases, with a total of 23,632 protein domains, 10,925 of which map to at least one human protein, and 7,369 map to at least one yeast protein. Functional feature information was collected for CDD domains from the "cddannot.dat" file located in the CDD FTP directory (ftp://ftp.ncbi.nih.gov/pub/mmdb/cdd), totaling 1,727 unique functional features. The non-overlapping set of human, non-synonymous disease mutations was created from the OMIM [29] and Swiss-Prot variant databases obtained from E-utilities and UniProt's FTP directory (http://www.uniprot.org/docs/humsavar) respectively. The resulting human mutation dataset consists of 32,653 mutations related to human diseases. The set of phenotypic yeast mutations was downloaded from SGD (the phenotype_data.tab database obtained from http://www.yeastgenome.org/download-data/curation/) and was filtered to exclude records without allelic information and records listing the phenotypic change as "normal," as these records refer to mutations with no phenotypic change. Finally, the yeast mutation database was manually curated to extract single point mutations and to ensure that each mutation record referred to a single point mutation. Mutation records referring to multiple mutations for a single phenotype were separated into multiple records. The final yeast mutational database is comprised of 1,490 unique mutations associated with phenotypic changes and is available upon request.

### Mapping mutations to protein domains

Hidden Markov models for protein domains from SMART, COG, CDD, and Pfam were built using multiple sequence alignments from CDD with the hmmerbuild tool (HMMer version 2.3.2) [30]. HMMer's hmmpfam tool was then used with the global option to search for complete domains in human proteins from the RefSeq and Swiss-Prot databases. Protein mutations were distributed to protein domain positions by using HMMer's alignment output for all domains aligning to the protein with an E-Value ≤ 0.001 and by assigning mutations on gap regions of the domain model to the last position before the gap. Each mutation was mapped only to the representative protein for each unique gene in the dataset. The methods for mapping domains to human proteins and disease mutations to their domain positions were previously described for our DMDM tool [17]. After distributing each mutation to all domains that map to the protein position in which the mutation was located, 4,283 human protein domains contained at least one disease mutation and 1,687 yeast protein domains contained at least one phenotypic yeast mutation.

### Determining the level of conservation at each domain position

For each column *j *in a protein domain multiple sequence alignment, we used the AL2CO [31] method to determine the entropies using the following formula

(1) $$H_{j}=-\sum_{i=1,20}p\left(a_{i,j}\right)\;\text{In}\;\left(p\left(a_{i,j}\right)\right),$$

where *p*(*a_i,j_*) is the frequency of amino acid *ai *at position *j*. We then estimated a threshold for identifying highly conserved positions by adding one standard deviation to the average of all AL2CO scores on all domain positions. As a result, a threshold of entropy less than or equal to 0.533 was used to determine conservation. The average entropy of each domain model was determined by estimating the mean of the entropy scores for all positions in the domain model.

### Estimating domain significance scores (DS-Scores)

**Figure 1:**
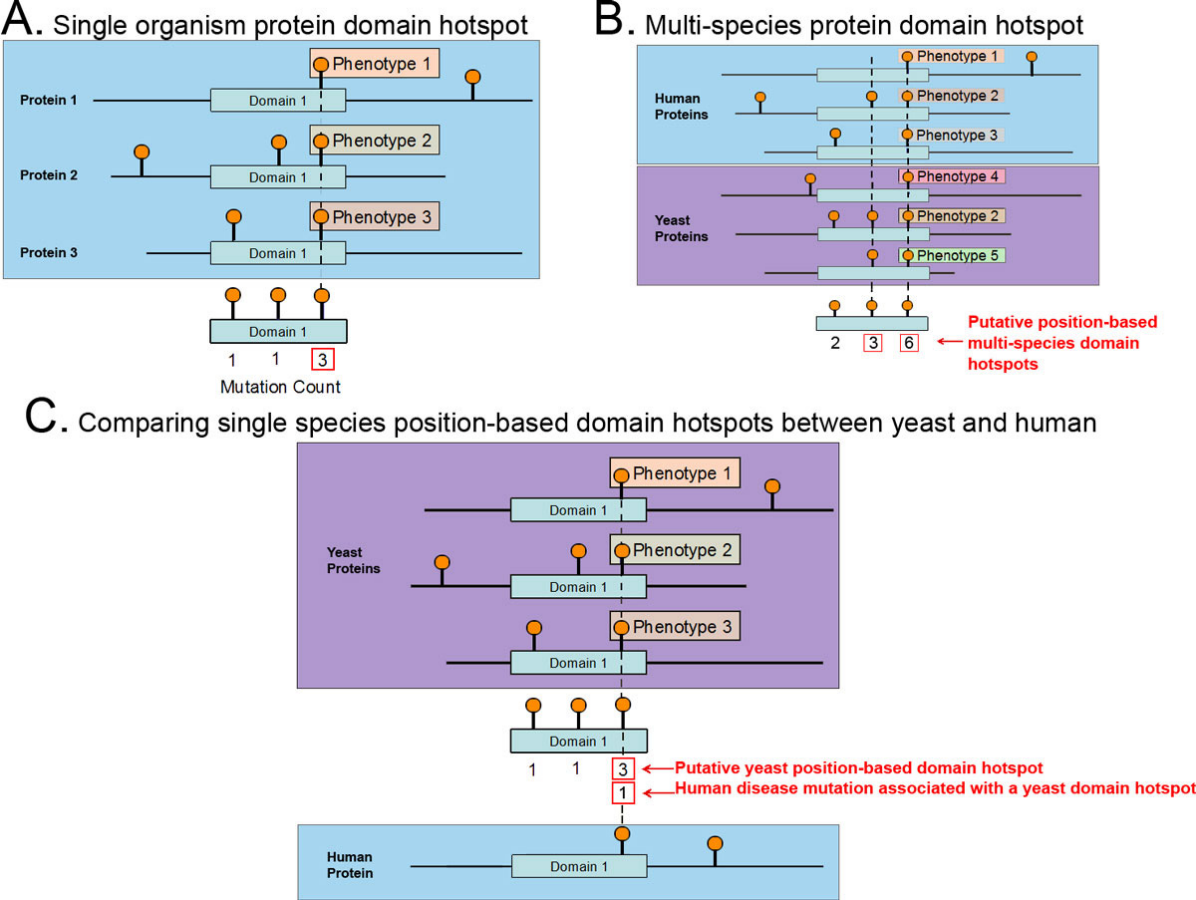
**Aggregating mutations using protein domains to form domain hotspots**. (A) Mutations from multiple proteins in a single organism sharing a common protein domain mapped to the domain level. These mutations have different phenotypes but can be shown to form a large cluster at a single position in the domain, indicating similarity. (B) Mutations from multiple organisms mapped to a protein domain. These mutations form a large cluster of mutations at the domain level despite having originated from different species. (C) A domain hotspot is formed using mutations from only organism 1, but mutations aligning to the same domain position from organism 2 do not have enough mutations to form a domain hotspot. However, some association can still be drawn due to the existence of a domain hotspot in organism 1 aligning to a mutation in organism 2.

We used a method previously developed by our group [18] to estimate the position-based and feature-based DS-Score for each position in the domain. Let *n *be the total number of mutations in the domain and let *L *be the number of possible positions in the domain. The random sample, X_1_, ... , X_L _consists of the numbers of mutations aggregated to the domain level from a single organism (Figure 1A) or from any number of organisms (Figure 1B) and let X_(1) _be the smallest of these X_i _, and X_(2) _be the next order of magnitude, ... , and X_(L) _is the largest X_i_. We used the probability mass function, P(X = x), to test whether the *n *mutations are randomly distributed into *L *positions. Finally, we defined our position-based DS-Score by the negative logarithm transformation of the binomial probability of observing a cluster of mutations of a particular size, given the total available positions in a domain and the total number of mutations observed. For consistency, when the number of mutations is equal in *m *positions, we assigned the same score to all *m *positions. Where k is the k^th ^order of the number of mutations at domain positions, we define the DS-Score as

$$\begin{array}{cccc} \text{DS - Score} & = & -log_{10}\left(P\left(\text{max}\left(x\right)\geq k\;and\;\text{max}\left(x\right)=x_{\left(L\right)}\right)\right) & \\ & = & \cdots \; & \\ & = & -log_{10}\left(P\left(\text{max}\left(x\right)\geq k\;and\;\text{max}\left(x\right)=x_{\left(L-m\right)}\right)\right) & \\ & = & -log_{10}\left(1-Pr{\left(x<k;Binomial\left(n,\frac{1}{L}\right)\right)}^{L}\right) & \end{array}$$

**Figure 2:**
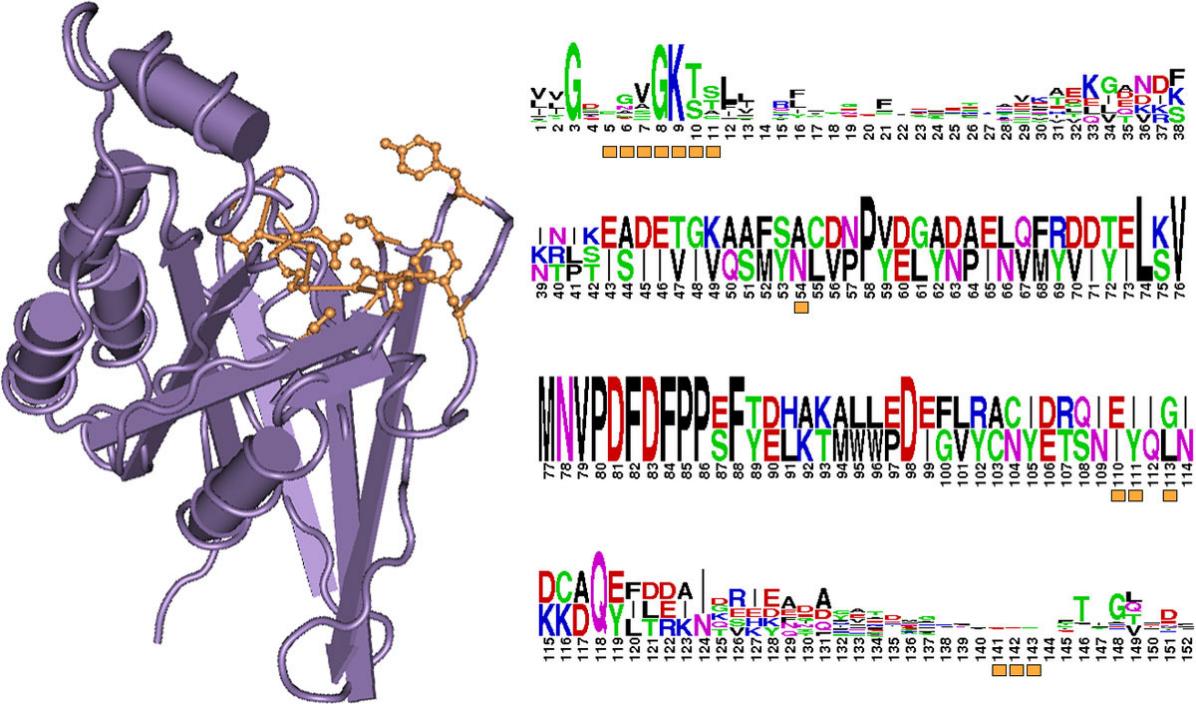
**Visual representation of position-based and feature-based domain hotspots**. The structure of the Ras-like protein domain (cd00882) of the human protein Cdc42 (PDB: 1CEE_A) is shown in purple (left). The sequence logo (generated using the WebLogo software [62]) represents a subset of the Ras-like protein domain from positions 1-152 (right). The functional feature residues corresponding to the GTP/Mg++ binding site at domain positions 5-11, 54, 110, 111, 113, and 141-143 are highlighted in orange on the structure (left) and are represented as orange boxes below each domain position in the sequence logo (right). Each of these functional feature protein domain positions will have a different position-based DS-Score (estimated based on number of mutations at each domain position) but equal feature-based DS-Score (estimated based on the maximum number of mutations found in any of the binding sites).

We defined domain hotspots using three levels of significance from the Fisher's scale of evidence for interpreting p-values as described by Yue *et al. *[32], namely p-values less than or equal to 0.025, 0.05, and 0.10. Using these three levels of significance, we derived three DS-Score thresholds of greater than or equal to 1.6, 1.3, or 1.0 corresponding to p-values less than or equal to 0.025, 0.05, and 0.10 respectively. In addition to position-based DS-Scores, which are based solely on the mutations found at a specific domain position, feature-based DS-Scores were created by distributing the largest position-based DS-Score for each functional feature to all other positions with the same functional feature annotation (see example in Figure 2). We define these functional feature sites as domain positions that have been manually annotated by CDD as having a known functional role (e.g. a DNA binding site or a flexible hinge region) [33]. Perl and R (http://www.r-project.org) were used to determine and distribute the DS-Scores for each domain position.

### Mutations and protein domain redundancy

Due to large domain superfamily hierarchies and duplicate domains from different sources, a single protein mutation can be mapped to many redundant domains. As a consequence, multiple domain hotspots can be identified on different domains that have originated from the same cluster of mutations. On one hand, to ensure that the DS-Scores and domain hotspots are estimated and identified for all protein domain models, all mutations in our analysis were distributed to all domains that map to the mutated protein position. The results of our analysis for each individual domain model, even for those domains from superfamily hierarchies and disparate sources for which we expect high redundancy, are available upon request. On the other hand, to prevent overestimating the number of hotspots shared between yeast and human, we designed a procedure for the non-redundification of domain hotspots. As a result, the numbers reported for all domain hotspots and multi-species domain hotspots originated from a unique set of mutation clusters. We excluded hotspots that originated from identical sets of mutations using the following method to select a unique representative domain for cluster of mutations. The representative domain is only used for visualization and internal calculations and does not affect the reported results. To select the representative domain for a mutation cluster, all domains were ordered alphanumerically from lowest to highest accession identifier. Preference is given to domains that are listed first within the list and were defined as root in the domain hierarchy. If none of the domains in the list is the root domain of a hierarchy, the representative domain is the first domain in the list that contain known functional annotated sites. In addition, when comparing hotspots using the multi-species DS-Score, the representative domain model is selected only among those that are shared among the species.

### Assessing the co-occurrence of human diseases and yeast phenotypes

The significance of overlapping human diseases and yeast phenotypes was calculated using a right-sided Fisher's exact test. The Fisher's test for each possible pair of human diseases and yeast phenotypic changes was estimated using the following values: the number of times the human disease and yeast phenotypic change (H and Y respectively) overlap, the number of times H overlaps with a yeast phenotype that is not Y, the number of times Y overlaps with a human disease that is not H, and the total number of overlaps between yeast and human. Human diseases and yeast phenotypes were considered to overlap if the associated mutations were found to localize at the same position of an identical domain. To avoid overestimation due to domain model redundancy, no protein mutation was counted more than once as overlapping with a single human disease or yeast phenotype.

## Results

### Distribution of mutations in protein domains

To study the distribution of phenotypically relevant mutations at the protein domain level, all proteins from the considered species, i.e., human and yeast, were aligned to one or multiple domains and their mutations mapped into these domains. First, we mapped all phenotypically relevant mutations from yeast and disease mutations from human to protein domains and analyzed the distribution of the location of these mutations with respect to their functional annotation and conservation over species as measured by the entropy of the domain site. Due to the redundancy and large hierarchies within the protein domain database, we observed that the 1,490 yeast and 32,653 human protein mutations were propagated to 11,016 and 323,840 domain mutations respectively (see Table 1). In Table 1, we also list the total number of domain sites with mutations and their breakdown into functionally annotated and conserved domain sites for yeast and human domains. We found a total of 8,186 domain sites mapped to 11,016 phenotypically relevant mutations in yeast, 3,992 of them with known function and 5,950 were conserved domain sites corresponding to 36% and 45% of the total number of yeast domain mutations. Similarly, we reported 323,840 domain mutations in 130,731 domain sites, with 58,096 (18%) corresponding to mutations in functionally annotated sites and 152,524 (47%) to mutations in conserved domain sites. Using Fisher's exact test, we estimated the enrichment of human and yeast mutations in functionally annotated and conserved sites. The results from our analysis produced p-values close to 0 for functionally annotated conserved sites in both human and yeast, which indicates a tendency for both the phenotypically relevant yeast mutations and the human disease mutations to be located at functional feature and conserved sites.

**Table 1:** Distribution of yeast and human mutations at functional and conserved sites

	Yeast	Human
Total phenotypically relevant protein mutations	1,490	32,653

Total phenotypically relevant protein mutations inside of domain regions	1,129 (76%)	24,301 (74%)

Total phenotypically relevant domain mutations	11,016	323,840

Domain positions with at least one mutation	8,186	130,731

Domain mutations at functional feature domain sites	3,992 (36%, p-value: ≈ 0)	58,096 (18%, p-value: ≈ 0)

Domain mutations at conserved domain sites	5,950 (45%, p-value: ≈ 0)	152,524 (47%,p-value: ≈ 0)

Counts for yeast and human mutations at functional feature and conserved sites. Fisher's exact test was used to compute the p-values for significant overlap of mutations with the functional and conserved sites.

### Transferring mutational information across species through protein domains

**Figure 3:**
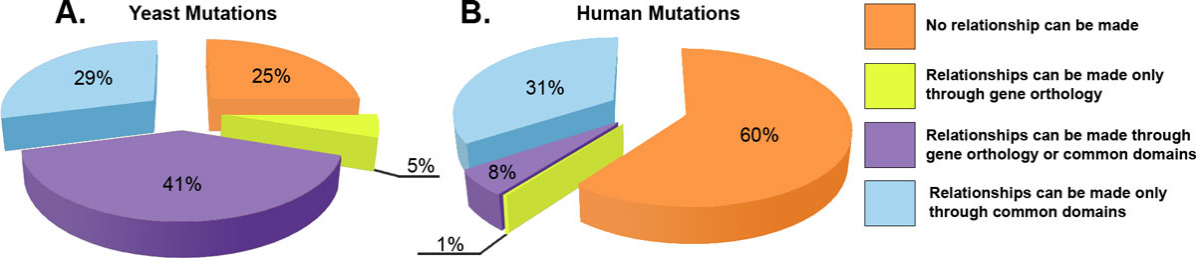
**Comparing orthologous genes and common protein domains between yeast and human**. Yeast and human protein mutations that can be related to mutations in other organisms through either orthologous genes from Homologene or through common protein domains. For both yeast (A) and human (B) mutations, each portion of the graph represents the mutations that could not be related to the other organism (orange), the mutations that could be related only through Homologene (yellow), the mutations that could be related using only common protein domains (blue), and the mutations that could be related using either method (purple).

When comparing the similarity between yeast and human genomes at the gene and domain levels, we found that the Homologene database can identify human orthologs for only 20% of the yeast genes, while 87% of protein domains found in yeast are also found in human. Most importantly, we quantified how many of the genetic alterations in the yeast and human mutation databases could possibly be related to the other species using either orthologous genes or common protein domains, as illustrated in Figure 3. We found that 435 (29%) of the yeast mutations and 10,187 (31%) of the human mutations can only be related to the other species using common protein domains and not orthologous genes, i.e., mutations located within a domain that is common between yeast and human but not located on orthologous genes. We also identified 610 (41%) yeast and 2,713 (8%) human mutations that could be related to the other species by means of either the common domain or orthologous gene. A small number of mutations, 68 (5%) from yeast and 310 (1%) from human were related to the other species using orthologous genes and not common protein domains, for example, a mutation outside of a protein domain region on an orthologous gene. The remaining mutations, 377 (25%) from yeast and 19,443 (60%) from human, could not be related between species using either method. This analysis was also performed using the orthologous gene information from OMA [34] and InParanoid [35] showing only minor variations in the results.

### Yeast and human mutations form hotspots at protein domain positions

**Figure 4:**
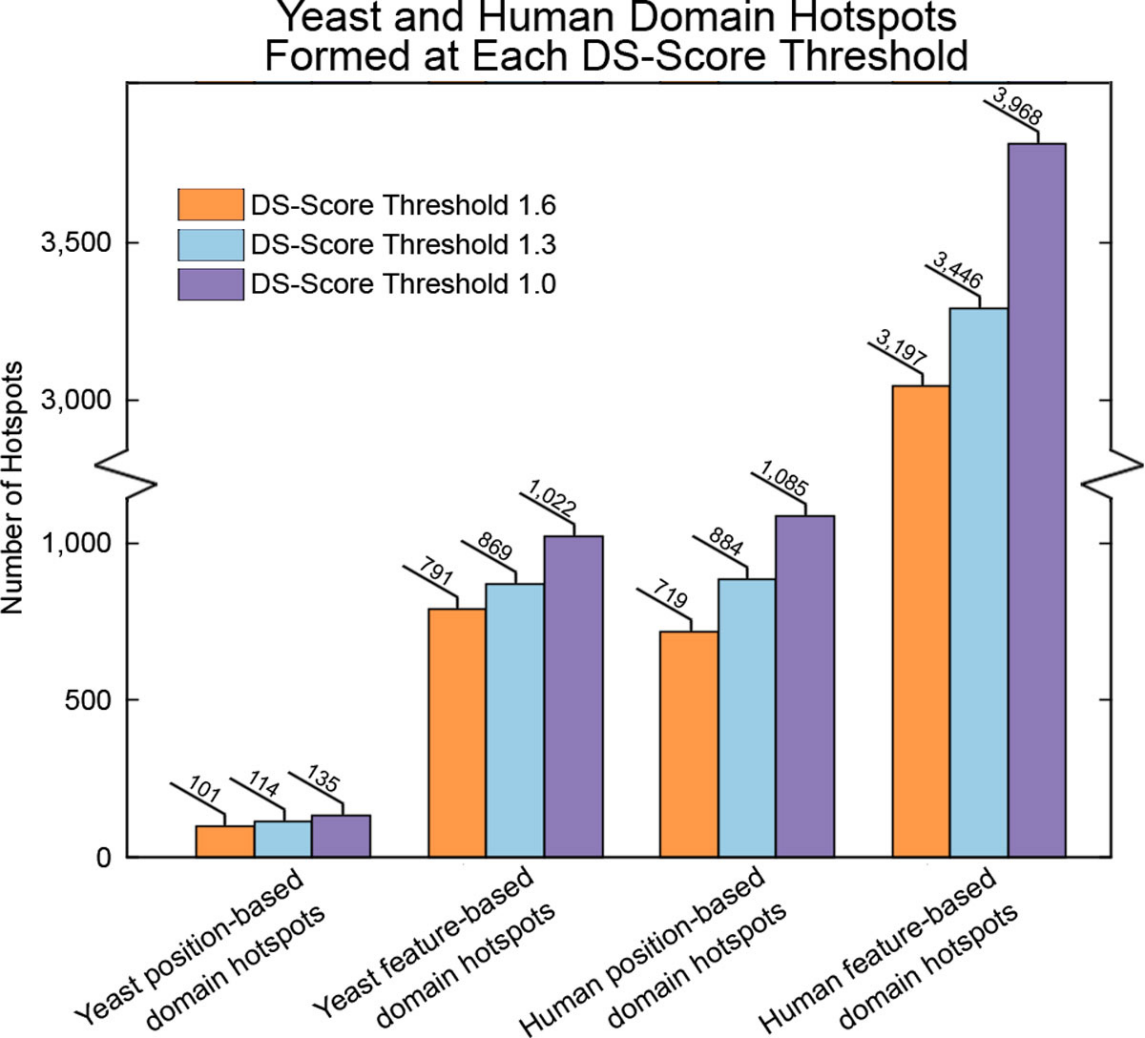
**Yeast and human domain hotspots formed at each DS-Score threshold**. Yeast and human position-based and feature-based hotspots formed DS-Score thresholds greater than or equal to 1.6, 1.3, and 1.0.

A statistical approach, the DS-Score, was used to identify clusters of mutations sharing the same domain position. The method can be used to cluster mutations from one (Figure 1A) or multiple species (Figure 1B) and clusters from one species can be associated with mutations from another species (Figure 1C). Three DS-Score thresholds were used in this study to identify significantly relevant clusters of mutations at the domain level, namely 1.6, 1.3, and 1.0, corresponding to p-values less than or equal to 0.025, 0.05 and 0.10 respectively. The number of domain hotspots found at each DS-Score threshold is shown in Figure 4. Most importantly, in this first analysis of phenotypically relevant yeast mutations at the protein domain level, we found that they cluster at specific domain positions forming 101, 114, and 135 position-based domain hotspots at DS-Score thresholds greater than or equal to 1.6, 1.3, and 1.0 respectively. The human mutations also form domain hotspots at these thresholds, resulting in 719, 884, and 1,085 position-based domain hotspots at DS-Score thresholds greater than or equal to 1.6, 1.3, or 1.0 respectively.

Using a variation of the DS-Score in which we emphasize the similarity of domain sites with the same functional annotation as depicted in Figure 2, we studied the feature-based domain hotspots in yeast and human. Our results show that yeast mutations on domains formed 791, 869, and 1,022 feature-based domain hotspots when using DS-Score thresholds greater than or equal to 1.6, 1.3, and 1.0 respectively, while human mutations yielded 3,197, 3,446, and 3,968 feature-based domain hotspots for these thresholds.

### Distribution of phenotypic and disease mutations at conserved and functional domain sites

**Figure 5:**
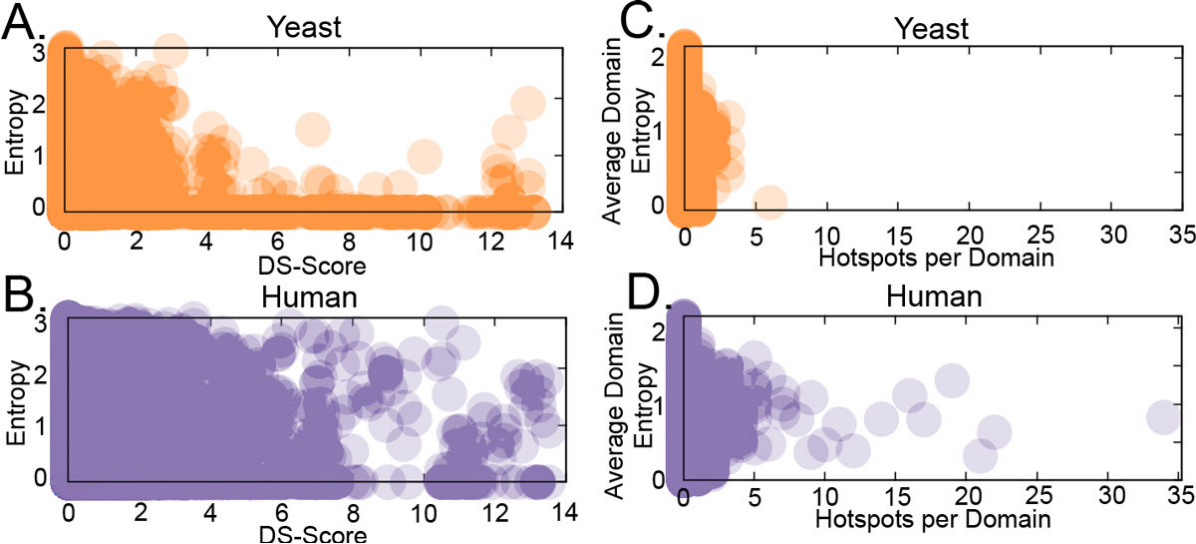
**Correlation between position-based DS-Score and Conservation (estimated by Entropy) for mutations in yeast and human**. The relationship between position-based DS-Score (using 1.3 as a threshold) and the conservation of the mutated domain position, as estimated by Entropy, is depicted for yeast (A) and human (B) domains. Additionally, the average entropy for each domain is compared with the number of domain hotspots (using 1.3 as a threshold) in yeast (C) or human (D). In this plot, the total number of domain hotspots is compared to the average Entropy of all positions in the domain model.

The distribution of DS-Score domain hotspots located on functionally annotated and conserved sites at each DS-Score threshold is presented in Table 2. Yeast position-based domain hotspots were located at highly conserved sites 55% of the time for the 1.6 threshold, 56% for the 1.3 threshold, and 50% for the 1.0 threshold. Additionally, the position-based domain hotspots for human were found at conserved sites 41%, 39%, and 40% of the time for thresholds 1.6, 1.3, and 1.0 respectively. Despite the enrichment of domain hotspots at conserved domain sites, the correlation between entropy and DS-Score is extremely low with Pearson correlation coefficients of -0.04 and -0.16 for human and yeast data, respectively (see Figures 5A and 5B). To assess the relationship between the number of domain hotspots per domain with respect to the sequence conservation of the domain model, we plotted the average position domain entropy versus their number of hotspots (see Figures 5C and 5D for yeast and human respectively). We found a correlation coefficient of -0.04 for both yeast and human, indicating that almost no correlation exist between the domain divergency and the number of domain hotspots it contains. We also analyzed how frequently the domain hotspots occurred at functional features sites. Our results show that 31%, 31%, and 33% of the position-based domain hotspots are located within annotated functional feature sites for phenotypically altering yeast mutations and 17%, 16%, and 16% for human disease mutations when using 1.6, 1.3, and 1.0 respectively as thresholds to define the domain position-based domain hotspots. When analyzing feature-based domain hotspots at these positions, our results indicated that 67%, 67%, and 63% of feature-based domain hotspots in yeast were localized at conserved sites using the 1.6, 1.3, and 1.0 thresholds respectively and 58%, 57%, and 57% for human feature-based domain hotspots respectively. Similarly, we found that 91% of the yeast feature-based domain hotspots, estimated using 1.6, 1.3 or 1.0 DS-Score thresholds, are located in functionally annotated sites. Likewise, for the human feature-based domain hotspots, we found that 81%, 78%, and 77% (as defined by the 1.6, 1.3, and 1.0 thresholds respectively) were localized within a functionally annotated domain site.

**Table 2:** Distribution of yeast and human domain hotspots at functional features and conserved sites

	Yeast (1.6)	Yeast (1.3)	Yeast (1.0)	Human (1.6)	Human (1.3)	Human (1.0)
Position-based domain hotspots at conserved sites	56 (55%)	64 (56%)	68 (50%)	295 (41%)	346 (39%)	431 (40%)

Feature-based domain hotspots at conserved sites	531 (67%)	582 (67%)	646 (63%)	1,859 (58%)	1,978 (57%)	2,265 (57%)

Position-based domain hotspots at functional features	31 (31%)	35 (31%)	44 (33%)	120 (17%)	137 (16%)	169 (16%)

Feature-based domain hotspots at functional features	721 (91%)	790 (91%)	931 (91%)	2,593 (81%)	2,691 (78%)	3,042 (77%)

The portion of human and yeast DS-Score hotspots at functional feature and conserved sites each threshold level, i.e., 1.6, 1.3, and 1.0.

### Phenotypically relevant mutations tend to cluster at domain positions in yeast and human

**Figure 6:**
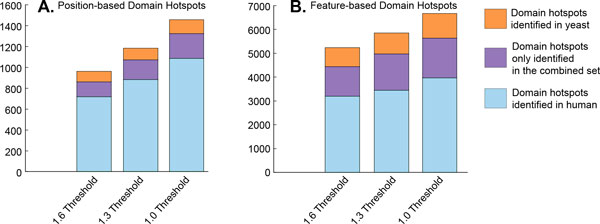
**Increase of domain hotspots by incorporating yeast mutational data**. The number of position-based (A) and feature-based (B) domain hotspots at the 1.6, 1.3, and 1.0 threshold. This figure visually represents the number of domain hotspots formed from human mutations (blue), the number of domain hotspots from yeast mutations (orange), and the number of new multi-species domain hotspots that did not reach significance in either the yeast or human analyses individually (purple).

Results from Table 3 on the analysis of domain hotspots for both species show that 103, 118, and 177 feature-based domain hotspots at the 1.6, 1.3, and 1.0 thresholds respectively and one position-based hotspot are common between yeast and human. The remaining yeast and human feature-based and position-based domain hotspots were unique to the organism in which they were found. One of the advantages of using additional species in the domain analysis of mutations is its potential for the identification of new domain sites of phenotypic relevance that become statistically significant when more annotated mutations are introduced. Thus, in addition to considering each organism's mutation datasets individually, our method was used to identify statistically significant clusters of a combined set of yeast and human mutations (as shown in Figure 1B). Table 4 outlines results for position-based and feature-based multi-species domain hotspots. In total, we found 861, 1,078, and 1,331 position-based multi-species domain hotspots for the 1.6, 1.3, and 1.0 threshold respectively. In addition, we found 4,243, 4,809, and 5,439 feature-based multi-species domain hotspots for the respective thresholds. We identified 143, 187, and 238 position-based multi-species domain hotspots, for the 1.6, 1.3, and 1.0 DS-Score thresholds respectively, that had not previously been identified when using human or yeast mutations independently. Similarly, we identified 1,243, 1,533, and 1,672 feature-based multi-species domain hotspots, for each DS-Score threshold level respectively, that had not previously been discovered when considering the human or yeast datasets independently. To illustrate the increase in the number of domain hotspots available for inference, Figures 6A and 6B depict the number of position-based and feature-based domain hotspots respectively that can be obtained using only human data, only yeast data, and by using a combined set of mutations from both organisms.

**Table 3:** Shared and unique domain hotspots between the yeast and human datasets.

	Domain Hotspot Count (1.6)	Domain Hotspot count (1.3)	Domain Hotspot Count (1.0)
Position-based domain hotspots only found in yeast	100	113	134

Position-based domain hotspots only found in human	718	883	1,084

Feature-based domain hotspots only found in yeast	688	751	845

Feature-based domain hotspots only found in human	3,094	3,328	3,791

Position-based domain hotspots shared between yeast and human	1	1	1

Feature-based domain hotspots shared between yeast and human	103	118	177

Position-based and feature-based domain hotspots for yeast and human sharing a common domain position at 1.6, 1.3, and 1.0 DS-Score thresholds.

**Table 4:** Multi-species domain hotspots

	Domain Hotspot Count (1.6)	Domain Hotspot count (1.3)	Domain Hotspot Count (1.0)
Total multi-species position-based domain hotspots	861	1,078	1,331

Total multi-species feature-based domain hotspots	4,243	4,809	5,439

Multi-species position-based domain hotspots not identified in yeast or human	143	187	238

Multi-species feature-based domain hotspots not identified in yeast or human	1,243	1,533	1,672

Position-based and feature-based domain hotspots identified by applying the DS-Score to a combined set of yeast and human mutations. This table shows the number of multi-species domain hotspots we can identify at each threshold, as well as the number of domain hotspots that were not previously identified when analyzing mutations from only yeast or only human.

### Linking domain hotspots with mutations across organisms

We analyzed the ability of domain hotspots to identify relevant mutations in other organisms. In particular, we focused on testing our ability to use yeast hotspots to identify disease mutations in humans. As depicted in Figure 1C, domain hotspots in one organism can be mapped to mutations in another organism through protein domains. When comparing domain hotspots in yeast to test if they could be mapped to any of the currently known human disease mutations, results in Table 5 show that 54 (53.5%), 56 (49.1%), and 65 (48.8%) of position-based domain hotspots in yeast at the 1.6, 1.3, and 1.0 thresholds can be mapped to at least one human mutation. The Fisher's exact test p-values for these results were 2e-26, 6e-25, and 5e-28 for each respective threshold level. Similarly, for yeast feature-based domain hotspots, we identified 562 (71.1%, p-value ≈ 0), 592 (68.1%, p-value ≈ 0), and 666 (65.2%, p-value ≈ 0) yeast domain hotspots that map to at least one human mutation. In addition, in Additional file 1, we list mappings of human domain hotspots into yeast mutational sites, also found to be statistically significant.

**Table 5:** Mapping of domain hotspots from yeast to known disease mutations in human.

	Domain Hotspot Count (1.6)	Domain Hotspot count (1.3)	Domain Hotspot Count (1.0)
Position-based domain hotspots in yeast	101	114	135

Feature-based domain hotspots in yeast	791	869	1,022

Position-based domain hotspots in yeast that hit at least one human mutation	54 (53.5%, p-value: 2e-26)	56 (49.1%, p-value: 6e-25)	65 (48.8%, p-value: 5e-28)

Feature-based domain hotspots in yeast that hit at least one human mutation	562 (71.1%, p-value ≈ 0)	592 (68.1%, p-value ≈ 0)	666 (65.2%, p-value ≈ 0)

Position-based and feature-based hotspots from yeast that could be associated with a human mutation through a common protein domain position were quantified (P-values from Fisher exact test are shown).

## Discussion

Our findings highlight the advantages of using protein domains to transfer information related to genetic mutations across species. We show that protein domain models provide a powerful framework for aggregating known phenotypically relevant mutation data across large evolutionary distances, i.e., from human and yeast. As a model organism, yeast is highly studied, well annotated, and easy to manipulate genetically. Thus, it is advantageous to transfer known information from genetic disruptions in yeast for analyzing human mutations. To infer relationships between mutations in different organisms, most studies use orthologous genes as reference to analyze mutations [36]. However, our analysis shows that yeast and human data share more common protein domains than they do orthologous genes. As a result, we show that mutations in both the yeast and human databases are better mapped across organisms when using shared protein domains than when using orthologous genes. For instance, we found that of the 40% of the human mutations that can be related to yeast, only 9% are through gene orthology while 39% can be related using a protein domain framework with an overlap of 5% of mutations that can be related by either domain or gene comparisons. This suggests that transferring mutational information by common protein domains not only vastly increases the number of mutations that can be transferred but also loses very few mutations that would have otherwise been transferred using only gene orthologs. The latter corresponds to, for instance, human disease mutations in genes for which there is a yeast ortholog but located outside a protein domain (only 1% of the human disease mutations in our analysis). Additionally, the domain approach allows the aggregation of mutations from multiple genes in each organism and the identification of relations between mutations located in non-orthologous genes by their functional annotation, which would normally be missed when analyzing the problem using a gene-centric approach.

Our study of phenotypically relevant mutations using a protein domain framework confirms that both yeast and human mutations show a significant tendency to fall within conserved and annotated functional protein domain sites. This is in agreement with the conclusions by Miller *et al.*[36]. In their study, the authors analyzed human disease mutations on seven disease-associated genes, cystic fibrosis transmembrane conductance regulator (*CFTR*), glucose-6-phosphate dehydrogenase (*G6PD*), neural cell adhesion molecule L1 (*L1CAM*), phenylalanine hydroxylase (*PAH*), paired box 6 (*PAX6*), the X-linked retinoschisis gene, and a gene associated with tuberous sclerosis (*TSC2*). From the study of mutations in these seven genes and their conservation across 20 organisms, including human, the authors concluded that these mutations are in highly conserved protein positions. Here, we reach similar conclusions, but we estimated conservation based on the protein domain models and not at the gene level. Additionally, our findings at the domain level are consistent with Mooney *et al. *[27]. The authors conducted a study on a set of 231 human genes with known disease mutations, showing that human disease mutations are statistically more likely to be localized within conserved or functionally relevant positions. To summarize, our domain-centric analysis confirms findings from gene-centric studies about enrichment of human disease mutations with respect to conserved and functionally annotated sites while identifying the same characteristics for phenotypically relevant mutations in yeast.

To analyze and compare yeast and human mutations we used the DS-Score method [18] and identified domain hotspots of human and yeast phenotypically relevant mutations. The DS-Score method was previously developed by our team to study human disease mutations and modified in this work to include mutations from both species resulting in the identification of multi-species domain hotspots. We also adapted the method for a multi-species analysis by removing redundant domain hotspots. As an extreme example of the effect of domain redundancy, a single cluster of mutations in the yeast *IRE1 *gene was propagated to over a hundred domains within the catalytic protein kinase domain family (cl09925 from the CDD database [21]), resulting in 120 domain hotspots having originated from the same cluster of domains. Similarly, domains from multiple sources (such as an identical domain from CDD and Pfam databases) could yield redundant domain hotspots counts. These redundant domain hotspots are correctly estimated and are of great relevance for the analysis of mutations in the context of individual domains. However, when comparing two species using redundant domain hotspots, if the cluster of mutations in the kinase family happens to be common to both species, we would reach the conclusion that there were 120 additional hotspots in common between yeast and human. To avoid overestimation of clusters of mutations that are aggregated at domain level, we defined domain hotspots as those having originated from a unique cluster of mutations and applied this method to the comparison of position-based and feature-based domain hotspots in both species. Using the catalytic protein kinase domain family as an example, each of the 120 domains in which the hotspot was found will retain this information, but only one representative hotspot, cd00180, which is at the top of the hierarchy in that kinase family from CDD [21], was considered for the final domain hotspot count for each species.

In this first-of-a-kind study of yeast mutations at the domain level, we demonstrate that phenotypically relevant mutations in yeast cluster at the domain level just as human disease mutations do, forming yeast and human domain hotspots that are the focus of this study. The hotspots in yeast present the same patterns as human domain hotspots in terms of enrichment at protein domain sites that are conserved and also in sites with known functional annotation. Neither the yeast nor human DS-Scores were found to correlate with conservation (as measured by entropy of the domain site), making the DS-Score method a complement to other methods for prioritization of mutations with putative phenotypic relevance such as SIFT [37], that use conservation as principal feature for their predictions.

We compared the newly found position-based and feature-based domain hotspots in yeast against those arising from human mutations. Given the limited number of mutations with known phenotypic association for each species, we expect the overlap between hotspots in both species to be low or non-existent. Indeed, the only position-based domain hotspot shared between yeast and human is at position 246 on PKc (cd00180), a kinase domain. Surprisingly, the number of hotspots in yeast and human sharing a common domain and functional annotation is 108, 114 and 177 when estimated using feature-based DS-score greater or equal to 1.6, 1.3, and 1.0 respectively. From this analysis, we concluded that the lower threshold provides a significant increase in the number of common feature-based domain hotspots found at the cost of including hotspots with lower significance. On the other hand, the similarity of mutation clusters between species increases when using feature-based DS-Score, a method designed to exploit the manually curated functional annotation provided by CDD [21]. The feature-based method provides some flexibility when comparing across species since it includes hotspots located in different positions for each species, as long as they are positioned in domain sites from the same domain and with identical functional annotation. For instance, using a feature-based threshold of 1.3, we were able to identify an additional 1,533 feature-based domain hotspots when considering DS-Scores generated from the combination of yeast and human mutations. To further underline the similarities between mutation patterns in both species, we analyzed the functional annotation of the domain hotspots and identified several that were unique to the species in which they were found but were localized on domain sites with identical annotation to sites that formed domain hotspots in the other species. Outlined in Table 6, a threshold greater than or equal to 1.3 was used to highlight examples of functional features that contained domain hotspots in both species. Domain positions annotated with the "Active site" functional feature were found to be identified as position-based domain hotspots in yeast 28 times and in human 32 times. Other functional features contained domain hotspots in both organisms as well. Among others, the "GTP/Mg2+ binding site" functional feature formed three yeast domain hotspots and seven human domain hotspots, and the "Substrate binding site," contained five yeast and 17 human domain hotspots.

**Table 6:** Selected functional feature sites containing domain hotspots in yeast and human

Functional Feature Name	Position-based Domain Hotspots in Yeast	Position-based Domain Hotspots inHuman
ABC transporter signature motif	1	2

Activation loop (A-loop)	4	99

Active site	28	32

ATP binding site	19	13

Ca2+ binding site	1	2

GTP/Mg2+ binding site	3	7

G1 box	3	6

Substrate binding site	5	17

Functional features found to contain position-based hotspots in human and yeast (using a DS-Score threshold of 1.3 to define the domain hotspots).

An example of a domain with several domain hotspots in human and yeast that highlights the advantages of using feature-based domain hotspots is the Ras-like GTPase domain (cd00882 from the CDD [21] database). While no position-based domain hotspots in the Ras-like GTPase domain from yeast and human are located at the same domain position, two hotspots are located at domain sites with the same functional annotation. The GTP/Mg++ binding site (highlighted in orange in the structure of the domain shown in Figure 2) contains position-based domain hotspots at position ten for the yeast mutations and at position five for the human mutations. The yeast hotspot in position ten is an example of mutations related at the domain level, originating from several genes, *ARF2*, *SEC4*, *GTR1*, and *NOG1 *that may not have been identified without analyzing mutations at the domain level due to the low sequence similarity of *NOG1 *(i.e. when using BLAST with E-Value < = 10^−3^). The yeast mutations in position ten of cd00882 in genes *ARF2*, *SEC4*, *GTR1*, and *NOG1 *were associated with increased sensitivity to cold [38], decreased rates of cytokinesis [39], decreased nutrient uptake [40], and inviability due to malformation of the large ribosomal subunit [41], respectively. In human, this domain position on the *ARL6 *gene contains a mutation associated with Bardet-Biedl syndrome type 3 [42] but not a domain hotspot. On the other hand, in a different position of the binding site, position five of the GTP/Mg++ binding region of the Ras-like GTPase domain contains a human hotspot that aggregates several positions in human genes that have been heavily studied due to their prominence in human diseases. This domain position corresponds to position 12 of both the human *HRAS *and *KRAS *genes, from which many mutations have been implicated in diseases such as Costello syndrome [43-46] and Congenital myopathy [47,48] and have also been found to be mutated frequently in somatic tumor samples from patients with follicular thyroid carcinoma [49], pancreatic carcinoma [50], and Schimmelpenning-Feuerstein-Mims syndrome [51], as well as bladder [52], lung [53], and gastric cancers [54]. While both *HRAS *and *KRAS *belong to the same protein family and are thus often implicated in the same studies, domain position five also aligns to position 38 of a gene from a different family, *GNAT1*, which is not similar in sequence to *HRAS *(i.e., *HRAS*-*GNAST1 *E-value of 0.53 using BLAST [55]) or *KRAS *(i.e., *KRAS*-*GNAST1 *BLAST E-value of 0.42). The *GNAT1 *mutation has been associated with congenital stationary night blindness [56]. Additionally, other mutations were found in the GTP/Mg++ binding pocket that were not members of position-based domain hotspots in either organism that we were able to identify using our feature-based domain hotspots. These mutations, sharing common functional annotation with position-based domain hotspots in both species, have been associated with autoimmune lymphoproliferative syndrome [57], somatic pilocytic astrocytoma [58], Noonan syndrome [59], and chylomiccron retention disease [60]. Thus, by extrapolating hotspots in human and yeast to common functional feature positions, we were able to identify a common functional disruption of the GTP/Mg++ binding pocket that causes different phenotypes when mutated in different genes sharing the same domain in the same organism as well as across organisms.

Furthermore, the domain-centric approach across species introduced here could also be extended to compare the particular phenotypes across organisms that can be related through mutations clustered with this approach, analogous to the phenotype similarities described by McGary *et al. *[12] using gene orthology. In Table 7, we show a preliminary analysis of this type of phenotype comparison by highlighting the human disease and yeast phenotypic annotations that most frequently co-occur using a domain-centric analysis. A complete list of all significant co-occurrences of human disease and yeast phenotypic changes can be found in Additional file 2. Interestingly, the most significant disease-phenotype co-occurrence was Wilson's disease, a human genetic disorder in which copper accumulates in tissues, with the yeast phenotype related to "Gain of function; metal resistance: increased." This yeast phenotype was derived from a study that analyzed a mutated copper-transporter gene, which resulted in a gain of function mutation that enabled the control of intracellular levels of cadmium through an enhanced cadmium efflux system [61]. While the relationship between the two phenotypes for the Wilson's disease example seems to be clear, for many of the significant co-occurrence of phenotypes found with our method, the relationship between the phenotypes is not apparent. An in-depth analysis of the molecular mechanisms and existing literature, as well as experimental validation will be needed to test and uncover novel hypothesis about molecular similarities between the two species.

**Table 7:** Human diseases and yeast phenotypic changes that co-occur at domain sites

Human Disease	Yeast Phenotypic Change	Number of Co-occurrences
Wilson's disease (WD) (OMIM:277900)	Gain of function; metal resistance: increased (PMID: 10743563)	6 (p-value: 2e-14)

Hereditary non-polyposis colorectal cancer type 2 (OMIM:609310)	Mutation frequency: increased (PMID: 16492773)	5 (p-value: 1e-13)

Susceptibility to Breast-Ovarian Cancer, Familial (OMIM:604370)	Reduction of function; protein/peptide accumulation: increased (PMID: 10218484)	4 (p-value: 4e-11)

Nemaline myopathy type 3 (OMIM: 161800)	Conditional; protein/peptide modification: absent (PMID: 16221887)	4 (p-value: 4e-11)

Familial hyperinsulinemic hypoglycemia type 1 (OMIM: 256450)	Reduction of function; replicative lifespan: decreased (PMID: 21931558)	8 (p-value: 1e-10)

Costello syndrome (OMIM:190020)	Inviable (PMID:17443350)	6 (p-value: 1e-09)

Methemoglobinemia, type 1 (OMIM:250800)	Reduction of function; heat sensitivity: increased (PMID: 19194512)	4 (p-value: 8e-09)

Crouzon syndrome (OMIM: 123500)	Resistance to chemicals: decreased (PMID: 17237519)	6 (p-value: 8e-09)

Kallman syndrome 2 with bimanual synkinesia (OMIM: 136350)	Resistance to chemicals: increased (PMID: 1715094)	4 (p-value: 4e-08)

Friedreich Ataxia (OMIM: 229300)	Protein activity: decreased (PMID: 19884169)	3 (p-value: 1e-06)

The top ten yeast phenotypic changes and human diseases that have a significant overlap at the domain level as determined by Fisher's exact test. Each disease co-occurrence is counted only once for each uniquely mapping mutation to avoid overestimation due to domain model redundancy.

## Conclusion and future work

This first-of-a-kind study demonstrates the aggregation of mutations from species spanning large evolutionary distances such as yeast and human. Using the DS-Score method as the framework for the integration of molecular characteristics, such as domain location and functional annotation of phenotypically relevant mutations, we were able to identify common mutation patterns from two distantly related species. The domain-centric approach introduced in this paper provides an ideal framework for the analysis of mutational data across species since the number of mutations that can be related from one species to another is much higher than what could be related through gene orthology. The feature-based method to compare mutations across species introduced here represents a unique way to integrate functional annotation of domains into the statistical analysis of mutations, shown here to be extremely advantageous for capturing similarities between mutations across distantly related species. This analysis also suggests that the approach is useful in relating phenotypes from yeast and human resulting from a particular pattern of mutations, such as being localized at the same domain position or functional site. We plan to perform a detailed analysis of the molecular basis of these related phenotypes. Hypotheses derived from this analysis have great potential for discovering new relationships between pathways and networks in both species. In addition, we plan to extend this study to other species to identify patterns across more closely related species including mouse, and to increase the number of known phenotypically relevant domain hotspots by including all mutational data available for a wide range of organisms.

## Competing interests

The authors declare that they have no competing interests.

## Authors' contributions

MGK designed the study, TAP performed the bioinformatics analysis, MGK and TAP prepared and analysed the data and drafted the manuscript. DP developed the algorithm for computing the mutation frequency significance thresholds. All authors read and approved the final manuscript.

## Supplementary Material

Additional file 1 **This file contains information related to the mapping of hotspots in human to mutations in yeast**. This file is in PDF format, and can be viewed using Adobe Reader or similar applications. Click here for file

Additional file 2 **This file contains the human disease and yeast phenotypic changes the co-occur at domain sites**. This file is in Microsoft Excel format, and can be viewed using Microsoft Excel or similar applications. Click here for file

## Declarations

The publication costs for this article were funded by the above grant.

This article has been published as part of *BMC Genomics *Volume 14 Supplement 3, 2013: SNP-SIG 2012: Identification and annotation of SNPs in the context of structure, function, and disease. The full contents of the supplement are available online at http://www.biomedcentral.com/bmcgenomics/supplements/14/S3
