# Supplementary material for: A protein domain-centric approach for the comparative analysis of human and yeast phenotypically relevant mutations
Source: BMC Genomics. 2013 May 28;14(Suppl 3):S5. doi: 10.1186/1471-2164-14-S3-S5 (PMC3665522; doi:10.1186/1471-2164-14-S3-S5)
Supplement: Additional file 1 — This file contains information related to the mapping of hotspots in human to mutations in yeast. This file is in PDF format, and can be viewed using Adobe Reader or similar applications. [file 1471-2164-14-S3-S5-S1.pdf]

Table S1: Mapping of hotspots from human to known disease mutations in yeast.
The significance p-value for human hotspots overlapping yeast mutations was calculated using fisher's exact test.

	**Domain Hotspot count (1.6)**	**Domain Hotspot count (1.3)**	**Domain Hotspot count (1.0)**
Position-based domain hotspots in human	719	884	1,085
Feature-based domain hotspots in human	3,197	3,446	3,968
Position-based domain hotspots in human that hit at least one yeast mutation	47 (6.5%, p-value: 9.26e-44)	51 (5.8%, p-value: 9.99e-45)	60 (5.5%, p-value: 3.00e-51)
Feature-based domain hotspots in human that hit at least one yeast mutation	428 (13.4%, p-value: ≈ 0)	454 (13.2%, p-value: ≈ 0)	500 (12.6%, p-value: ≈ 0)
